# Stabilizing lateral ankle instability by suture tape – a cadaver study

**DOI:** 10.1186/s13018-019-1218-6

**Published:** 2019-06-13

**Authors:** Heinz Lohrer, Giuseppe Bonsignore, Nadja Dorn-Lange, Lu Li, Albert Gollhofer, Dominic Gehring

**Affiliations:** 1 ESN – European Sportscare Network, Borsigstraße 2, 65205 Wiesbaden, Germany; 2 Lilium Klinik, Borsigstraße 2, 65205 Wiesbaden, Germany; 3 Institut für Sport und Sportwissenschaft, Albert-Ludwigs-Universität Freiburg, Schwarzwaldstraße 175, 79117 Freiburg, Germany; 4 Institut für funktionelle und klinische Anatomie, Johannes Gutenberg-Universität Mainz, Johann-Joachim-Becher-Weg 13, 55128 Mainz, Germany

**Keywords:** Ankle, Lateral ankle instability, Suture anchor, Suture tape, Augmentation, Cadaver study

## Abstract

**Background:**

Suture tape is a recent development to augment a Brostrom repair at least during the healing phase of the native tissues used for stabilization of the lateral ankle ligaments. The purpose of this study was to evaluate whether suture tape is an effective mechanical stabilizer against anterior talar drawer in a cadaver experiment when tested with a validated arthrometer.

**Methods:**

Different stability conditions were created in 14 cadaveric foot and leg specimens. Following anterior talofibular ligament (ATFL) dissection, isolated suture tape ATFL reconstruction was compared to the unaltered specimens, to the condition with ATFL cut, to the ATFL plus calcaneofibular ligament (CFL) cut conditions, and to the ATFL, CFL, and posterior talofibular ligament transected specimens. Three-dimensional bone-to-bone movement between fibula and calcaneus were simultaneously recorded using bone pin markers. Anterior translation was analysed between 20 and 40 N anterior talar drawer load, applied by an ankle arthrometer. Test conditions were compared using non-parametric statistics.

**Results:**

Dissection of ATFL increased anterior talar drawer in arthrometer and bone pin marker analyses (*p* = 0.003 and 0.004, respectively). When the CFL was additionally cut, no further increase of the anterior instability could statistically be documented (*p* = 0.810 and 0.626, respectively). Following suture tape reconstruction of the ATFL, stability was not different from the unaltered ankle (*p* = 0.173).

**Conclusions:**

Suture tape augmentation of the ATFL effectively protects the unstable anterolateral ankle in the sagittal plane. The CFL does not seem to stabilize against the anterior talar drawer load.

## Background

Ankle sprain is the most common injury in the physically active population and development to chronic ankle instability (CAI) is frequent [1]. About one out of five osteoarthritic ankles results from lateral ankle sprain [1]. The role of the mechanical component in CAI is a matter of ongoing debate [2]. Most studies originating from a kinesiology or sport scientific perspective do not report “a definitive association of ankle laxity with CAI” [3]. In the orthopaedic literature, however, CAI and mechanical ankle instability (MAI) are often used interchangeably [4–8]. When MAI exceeds a certain amount and functional deficits cannot adequately be restored by conservative approaches, operative interventions have to be taken into consideration. In principle, tenodeses, anatomic reconstructions, and combined procedures are used to stabilize against ligamentous lateral ankle instability [9, 10]. Repair using local tissue has been shown to effectively stabilize the lateral ankle joint [8, 11]. Suture tape is a recent development to augment a Brostrom repair at least during the healing phase of the native tissues used for stabilization of the lateral ankle ligaments [12, 13]. Although there are concerns for progressive elongation of suture-tape, unclear long-term stability (longevity of mechanical stability), and unexpected complications such as foreign body reaction, this procedure is increasingly used for various ligament reconstructions.

A clinical study examined the effectiveness of suture tape to stabilize the ankle against a manually performed anterior drawer test. Results led to the conclusion that using an additional suture tape might be favoured compared to an isolated modified Brostrom repair [14]. Until now, only three cadaver experiments were published to demonstrate the stability of anterior talofibular ligament (ATFL) suture tape augmentation [13, 15, 16]. These experiments either investigated load to failure of the isolated ATFL [13], or rotation of the tibia, respective to the calcaneus [15, 16]. These experiments were designed to only load the ATFL (or the respective reconstructions), and specimens were therefore rigidly fixed to the testing apparatuses. In contrast, our ankle arthrometer applied only low load to the unconstrained heel in an anterior direction. Due to the complex anatomy of the hindfoot, this load is transferred into a complex motion of the calcaneus and the talus with respect to the fixed leg. In contrast to previous cadaver experiments, we therefore aimed to more functionally test the whole lateral ankle ligament complex in a clinically relevant situation (anterior talar drawer), its contribution to stability, and the effect of suture tape ATFL augmentation (Table 1). In previous experimental approaches, the loading of the ankle joint complex was quantified externally with specific measurement devices, which can be applied only to cadavers. Until now, no study compared the specific motion between the interacting bones (fibula and calcaneus) and its functional representation in an ankle arthrometer, which is validated for experimental and clinical use in a native condition, following incremental lateral ligament dissection, and following suture tape application.

**Table 1:** Overview of the literature presenting experimental suture tape testing

Authors	Year of publication	*N*	Testing device	Analysed motion	Measured variable	Result
Viens et al. [13]	2014	3 × 6	Dynamic tensile testing machine (ElectroPuls E10000, Instron Systems, Norwood, Massachusetts)	Anterior talar drawer	Load to failure, stiffness	Brostrom plus suture tape augmentation vs. intact: no significant difference
Schuh et al. [15]	2016	3 × 6	858 Mini Bionix (MTS Systems Corporation, Eden Prairie, MN, USA	Internal rotation	Axial rotation (tibia vs. hindfoot)	Suture tape augmentation (angle at failure, failure torque) superior
Willegger et al. [16]	2016	2 × 6	858 Mini Bionix (MTS Systems Corporation, Eden Prairie, MN, USA	Internal rotation	Axial rotation (tibia vs. hindfoot)	Suture tape augmentation: similar biomechanical stability compared to an intact native ATFL (torque at failure, angle at failure)
Lohrer et al. (present study)	2019	14	Ankle arthrometer	Anterior talar drawer	Displacement	Suture tape ATFL reconstruction vs. intact: no significant difference

The purpose of this study was to evaluate the effect of ATFL suture tape augmentation using a previously validated anterior talar drawer arthrometer in a cadaver experiment. Additionally, the spatial bone-to-bone movement was examined by motion analyses using intraosseous markers.

## Methods

This investigation is part of a larger research project, which also aims to experimentally evaluate and validate the ankle arthrometer for assessing ankle laxity (anterior talar drawer).

The local Ethics Commission approved the study.

### Specimens

Fourteen fresh-frozen cadaveric above-knee amputated foot and leg specimens, obtained from four female and four male donors (median age = 78.5, range = 66–91 years), were thawed. We repeatedly irrigated the specimens with saline during dissection and testing to prevent desiccation. An experienced anatomist dissected all specimens down to the lateral ankle ligaments and capsule creating a square (5 × 5 cm) skin and subcutaneous window centered over the tip of the lateral malleolus. Lateral ankle ligaments were then identified, inspected for completeness, and were manually tested for anterior talar drawer and talar tilt. All ankles were stable and had no relevant ankle or hindfoot pathology. Following the experiments, the specimens were dissected to the bone to ensure that there was not any bony impingement or bone abnormality that could skew the data.

### Test procedure

Initially, the unaltered specimen was placed on the ankle arthrometer and was tested. Five repeated trials were registered and the mean was calculated and used for further analyses. Then, the ATFL was cut and the testing procedure was repeated. Thereafter, the ATFL was reconstructed by suture tape (InternalBrace^TM^, Arthrex, Naples, FL) and the measurements were repeated. Then, the CFL was cut while ATFL tape augmentation was unaltered and measurements were repeated. In the next step, tape augmentation was cut and the measurements were repeated. Finally, the posterior talofibular ligament (PTFL) was cut and measurements were completed to test for maximum translation.

This procedure enabled registration of stability/instability data relative to sequentially increasing lateral ankle ligament instability and to the effect of ATFL suture tape augmentation relative to isolated ATFL and to combined lateral ligament transections.

### Surgical approach

**Fig. 1:**
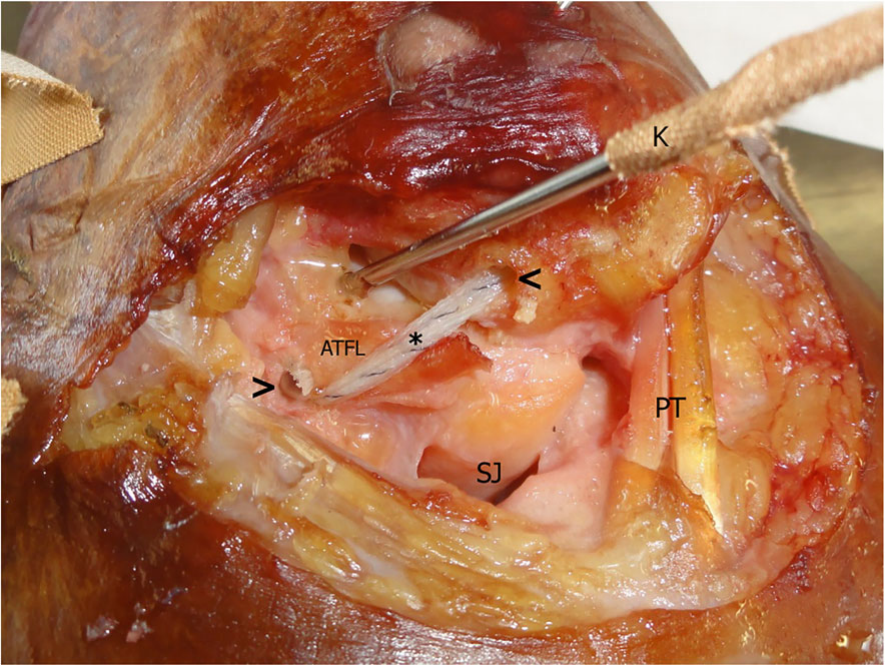
Example of suture tape reconstruction after dissection of an anterior talofibular ligament (ATFL). Left ankle specimen. Suture tape is fixed with anchors in the anterolateral distal fibula (<) and talus (>). The suture tape (asterisk) covers the original course of the ATFL. PT peroneal tendons, SJ subtalar joint, K K-wire

In our setup, the implantation of the suture tape was performed as an isolated ATFL reconstruction. The transected ATFL was not repaired. An experienced orthopaedic surgeon performed the procedure according to the manufacturer’s guidelines (fibula to talus) [12] with the manufacturer’s single-use instruments and implants (InternalBrace^TM^, Arthrex, Naples, FL). A 2.7-mm drill hole was created at the anterior margin of the fibula 2–3 mm lateral to the middle of the visible ATFL origin. After tapping, a 3.5-mm knotless anchor, preloaded with suture tape, was inserted (Fig. 1). The talar ATFL insertion was then identified and drilled in a 45° posteromedial direction with a 3.4-mm drill. After tapping, a 4.75-mm knotless anchor was preloaded with the free ends of the suture tape that was already attached to the fibula. According to implantation guidelines, it was inserted creating moderate tension to the tape [12].

### Ankle arthrometer

**Fig. 2:**
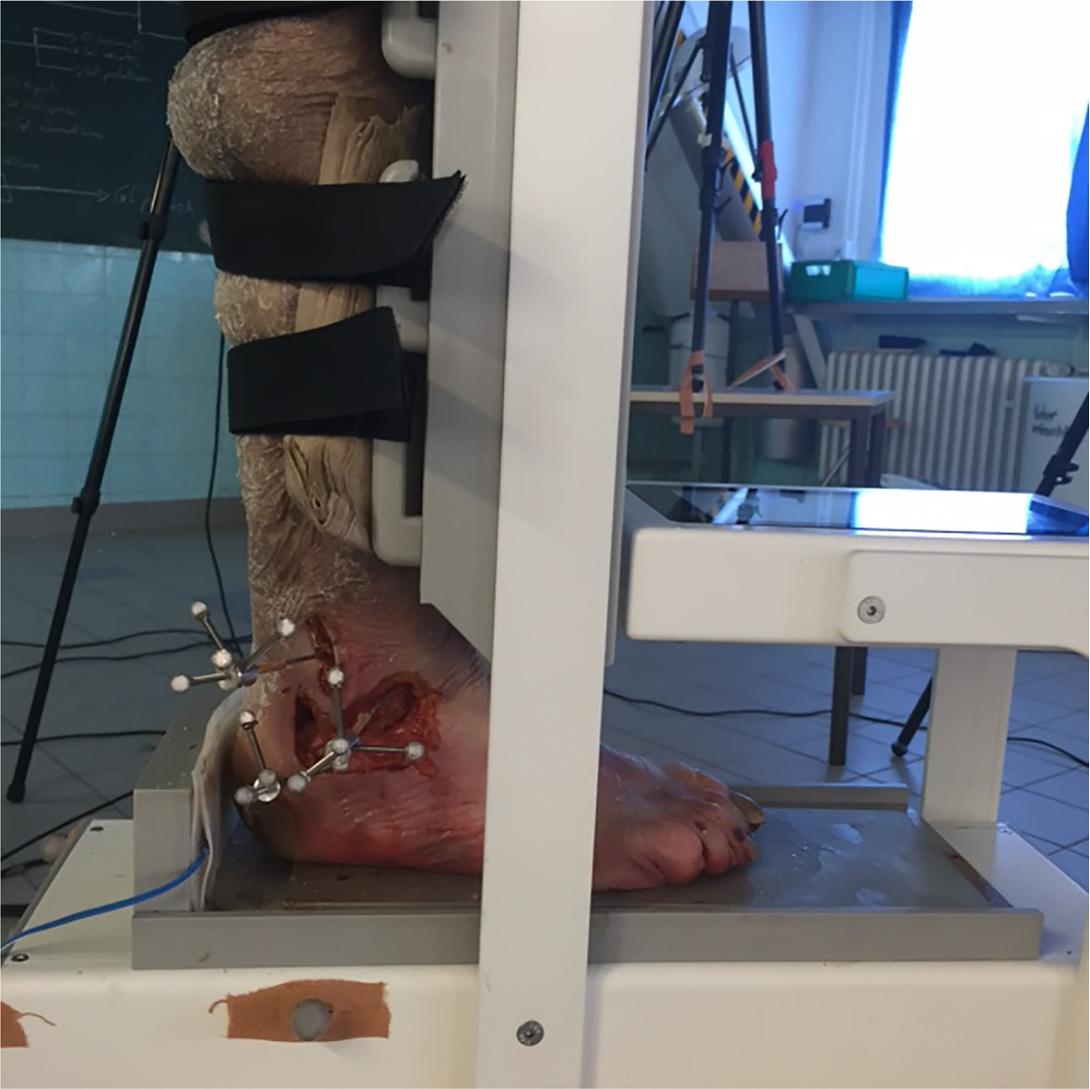
Photograph, demonstrating the test setup. The leg is fixed to the shin pad of the arthrometer with straps. The footplate induces anterior translation load via the heel pad. Fibula, talus, and calcaneus are equipped with K-wires, which carry 3D Cartesian coordinate systems with spherical reflective markers

Non-invasively, the anterior drawer was induced and measured with an arthrometer. The technical principle of the construction and the previous validation process has been described in detail elsewhere [2, 17–20]. In short, the ankle arthrometer is featured by a sliding plantar plate with heel pad which induces anterior translation of the foot relative to the leg, which is fixed to an anterior shin pad (Fig. 2).

Following a pilot study, the ankle arthrometer was modified for the current study. The drawer plate containing the foot of the arthrometer was additionally equipped with a ball bearing plate to reduce friction between the specimen’s sole and the drawer plate.

The ankle arthrometer induces an anterior drawer with a velocity of 8 mm/s. The arthrometer applied load from 0–80 N and displacement was simultaneously recorded from the ankle arthrometer and kinematically (bone pin coordinate system). The displacement between 20 and 40 N was calculated from the load-displacement curves. This interval was selected following results from previous work, indicating that ankle instability can best be differentiated in that low load region, representing the linear slope of the force-displacement curve [2, 18–20]. A possible slack length of the suture tape should be compensated for within the first 20 N of load application and therefore might not affect the measurements.

### Biomechanical testing

Three-dimensional bone-to-bone displacement during the anterior drawer within the ankle arthrometer was measured simultaneously and directly for the different test conditions. The methodology is described in detail in a further manuscript [21]. In short, using a motion analysis system (Vicon Motion Systems, Oxford, UK) with 11 cameras, the bony movement of the fibula, talus, and calcaneus were tracked at 200 Hz. For this purpose, 1.5 mm K-wires were drilled into the distal anterolateral fibula, the talar neck, and the lateral calcanear facet. Each K-wire was equipped with a frame, containing four 6 mm spherical reflective markers which defined a 3D Cartesian coordinate system (Fig. 2). These technical coordinate systems tracked the three-dimensional motion of each bone when performing the arthrometer testing. The relative motions between the fibula, talus, and calcaneus were calculated following the recommendations of the International Society of Biomechanics for the ankle joint using a joint coordinate system approach [22]. This approach allows for calculating three-dimensional rotations and translations of bone segments and had previously demonstrated its potential in detecting anterior translation instability of the ankle [23].

### Statistical analyses

Statistical analyses were performed using the SPSS statistical package 22.0 for macOS (IBM Inc.) and MATLAB-Software (MathWorks Inc.). As evaluation with the Kolmogorov-Smirnov test indicated that most of the variables were not normally distributed, dependent non-parametric comparisons were made for further analyses. Therefore, dependent descriptive non-parametric comparison (median and mean deviation) was made. For post hoc testing between two separate conditions, the Wilcoxon sign-rank test was applied and adjusted according to the Bonferroni-Holm correction procedure. For all analyses, the overall level of significance was defined at *p* < 0.05. Descriptive results are reported as median ± mean deviation around the median. Pearson correlations were calculated between all arthrometer data for all conditions (ligaments intact, ATFL cut, ATFL and CFL cut, ATFL cut and internal brace, ATFL and CFL cut and internal brace, ATFL and CFL and PTFL cut) and the 3D kinematic data (calcaneus vs. fibula).

## Results

Macroscopically, there was no failure of the suture tapes or the anchor fixations during the testing procedures.

**Fig. 3:**
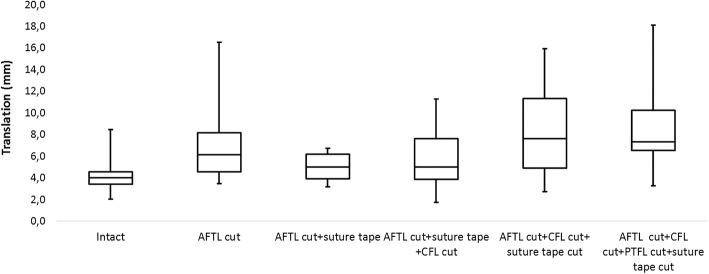
Box plot diagram, demonstrating the results of the ankle arthrometry relative to the specific test conditions. The horizontal lines within the boxes represent the median values. The lower and upper border of the boxes indicate the 25th and 75th percentile. The ends of the vertical bars represent the smallest and largest observed values. Respective *p* values are presented in Table 2. ATFL anterior talofibular ligament, CFL calcaneofibular ligament, PTFL posterior talofibular ligament

Ankle arthrometry (Fig. 3): Measured with the ankle arthrometer, the median anterior translation of the intact specimens was 4.0 ± 1.1 mm. Following the dissection of the ATFL median displacement increased to 6.1 ± 2.9 mm (*p* = 0.003) and to 7.6 ± 3.6 mm when the CFL was additionally cut (*p* = 0.001). With suture tape reconstruction, anterior translation was 5.0 ± 1.2 mm and 5.0 ± 2.1 mm, when solely the ATFL or both, ATFL and CFL were cut, respectively. Both reconstructed conditions were not different from the intact condition (*p* = 0.173 and 0.078, respectively).

Bone pin measurements (Table 2): Compared to the intact condition (0.2 ± 0.6 mm), anterior translation increased following the dissection of the ATFL (2.4 ± 4.9 mm; *p* = 0.004) and additionally the CFL (2.9 ± 6.5 mm; *p* < 0.001). Adding the suture tape reduced the anterior translation to 0.9 ± 1.0 mm when solely the ATFL was cut (*p* = 0.173) and to 0.7 ± 1.3 mm when additionally the CFL was cut (*p* = 0.007).

**Table 2:** Median distance as simultaneously measured with the ankle arthrometer and with bone pins between 20 and 40 N anterior talar drawer for the sequential ligament dissection and reconstruction compared with the intact ankle. The ankle arthrometer measures the anterior translation of the calcaneus with its surrounding soft tissue against the leg. The bone pin measures represent the translation between fibula and talus

	Ankle arthrometer					Bone pin measures (calcaneus vs. fibula)				
	Median (mean deviation) [mm]	Δ to intact [mm]	Δ to intact [%]	*P* value vs. intact	Bonferroni-Holm-corrected threshold	Median (mean deviation) [mm]	Δ to intact [mm]	Δ to intact [%]	*P* value vs. intact	Bonferroni-Holm-corrected threshold
Intact	4.0 (1.1)					0.2 (0.6)				
ATFL cut	6.1 (2.9)	2.1	53	*0.003*	*0.012*	2.4 (4.9)	2.17	1033	*0.004*	*0.012*
ATFL cut + suture tape	5.0 (1.2)	1.0	25	0.173	0.405	0.9 (1.0)	0.64	304	0.173	0.306
ATFL cut + suture tape + CFL cut	5.0 (2.1)	1.0	25	0.078	0.312	0.7 (1.3)	0.50	238	*0.007*	*0.035*
ATFL cut, CFL cut, suture tape cut	7.6 (3.6)	3.6	90	*0.001*	*0.006*	2.9 (6.5)	2.66	1266	*< 0.001*	*0.001*
ATFL cut, CFL cut, PTFL cut, suture tape cut	7.3 (3.1)	3.3	82	*0.001*	*0.006*	6.1 (6.0)	5.93	2823	*< 0.001*	*0.001*

Significant values are presented in italics

The correlation for the comparison between the exact motion of interacting bones (fibula vs. calcaneus) and its functional representation in the ankle arthrometer for the 20–40 N anterior talar drawer load was *r* = 0.851 (*p* < 0.001).

## Discussion

This study clearly demonstrates that an experimentally created anterolateral ankle instability (ATFL dissection) can effectively be reduced to anterior talar drawer baseline values by suture tape implantation. When compared to the intact condition and for the tested load (20–40 N), the CFL does not seem to play a relevant role for stabilizing against the anterior talar drawer, if the ATFL is reconstructed by suture tape. This behaviour is interesting, because our experimental setup tested for anterior translation, but the CFL is thought to protect mainly against ankle inversion [24, 25]. Because a large portion of patients with chronic ankle instability have insufficiency of both ligaments, further study regarding the suture tape ATFL and CFL reconstruction can be important and interesting.

Anatomic repair/reconstruction is currently the mainstay for operative treatment of MAI [10, 14, 26, 27]. In the last decade, anchor systems have been introduced to secure transplants [28] or as a knotless fixation for sutures [8]. Suture anchors are most important for the evolving arthroscopic Brostrom techniques [29, 30]. The stability of anchor systems has been tested in cadaver studies, but the strength of these repairs was inferior to native ATFL [31–34]. Recently, suture tape techniques for augmentation of the ATFL were described [12, 35] and are proposed at least for patients with generalized ligamentous laxity [26], athletes, or patients with poor local tissue quality, e.g., following failed previous repair or reconstruction [13, 14].

An advantage of these techniques is the additional mechanical stability, provided by the suture tape which is securely fixed to cover the ATFL from its anatomic fibular origin to its talar insertion [13, 15, 16]. So, safer and faster rehabilitation is thought to be possible [12, 14, 27]. Compared with tenodesis, no “donor site morbidity” can occur [35].

In a clinical study, young females with MAI underwent an isolated minimally invasive suture tape augmentation of the ATFL and CFL. After a minimum follow-up of 2 years, “91.2% achieved satisfactory functional results” [35]. In another study, patients “were able to quickly return to activity and sports” [14].

A disadvantage of the suture tape procedure is the “possibility of progressive elongation over time” [35]. But otherwise, this could turn into an advantage by “allowing the natural tissues to progressively strengthen” [12]. An uncontrolled study described “favourable” short-term outcome [27]. The most critical point for the suture tape implementation until now is that no long-term follow-up is available to determine effects and side effects.

There are few reports to demonstrate the stability of the suture tape augmentation in cadaver experiments. Previous cadaver experiments tested load to failure of suture tape augmentation (Table 1). In these experimental setups, the talofibular joints were extensively dissected and isolated. The specimens were rigidly fixed in the test apparatuses while internal rotation load was applied to the calcaneus relative to tibia/fibula [13, 15, 16, 31, 32]. That highly standardized procedure tries to apply the load exactly in the direction of the suture tape. Contrasting to this, our investigation was performed following only minimal dissection of the specimens to mimic the specific motion of the involved bones during anterior talar drawer. The measurements were performed between 20 and 40 N anterior talar drawer load with our arthrometer which was developed, validated, and used for testing in the clinical situation [2, 17–20]. However, our arthrometer does not evaluate the coronal plane motion (varus/valgus) of the ankle, but this motion has been analysed in a different approach within the same project [25].

Discussion is open whether additional CFL repair or augmentation is necessary. The effect of suture tape ATFL augmentation on talar tilt has not been addressed in cadaver studies (Table 1). However, a cadaver study demonstrated no difference in initial varus instability between isolated ATFL and combined CFL and ATFL repair with a Brostrom-Gould procedure [36]. This conclusion is also supported by an uncontrolled clinical study in an athletic population using a modified Brostrom procedure without CFL reconstruction [37]. When compared to the intact condition and for the tested load (20–40 N), the CFL does not seem to play a relevant role for stabilizing against the anterior talar drawer load, if the ATFL is stabilized by suture tape (Table 2). Recently, a minimally invasive technique was presented to anatomically augment both ATFL and CFL [35]. To further elucidate the role of the CFL, we addressed specifically the frontal plane instability in another approach [25].

Limitations to this study could be the higher age of our donors resulting in reduced bone and ligament quality. However, due to intraindividual comparisons of the different testing conditions, these differences were not likely to influence the results. Ankle degeneration could also influence the mobility during our tests, but all our specimens were free from degenerative findings. To get information about the isolated suture tape effect, we performed no Brostrom repair and therefore the individual quality of the lateral ankle ligaments does not play a role for the comparisons. However, this procedure is different from the standard clinical setting. We do not suggest that this study should be interpreted to promote isolated suture tape reconstruction to replace the modified Brostrom repair. It can be expected that an additional repair of the local tissue by a Brostrom procedure would lead to even more stability if the balance of load between the suture tape and the repair were properly set. Interestingly, isolated suture tape augmentation without addressing the ligaments has been recently shown to be effective in a clinical MAI study [35]. An additional limitation is that the cadaver study design only determines the response to the tape at the time of implantation. The stability measures would likely change over time due to wear out of the tape. Furthermore, this study evaluates the lateral ankle instability executed by an anteriorly applied load to the posterior calcaneus. The lateral ankle ligaments, however, stabilize the hindfoot in more complex and three-dimensional ways. That behaviour should be subjected to further analyses.

In principle, differences between ankle arthrometer and bone pin measurements are to be expected. The arthrometer provides an external instrumented measure, while the bone pins approach directly assesses the relative bone-to-bone movement between calcaneus and fibula. However, both measures demonstrated a strong relationship (*r* = 0.851). Our previous validation studies for the ankle arthrometer were done in a 2D radiographic approach in cadavers [19] or by in vivo comparison with a manual stress testing [2, 18]. As we were interested in having the most possible accuracy for the spatial bone-to-bone interaction, and to further evaluate the functional representation of the bone-specific movements, we decided to also investigate the ankle arthrometer against measurements obtained from a very precise biomechanical measuring tool. Consequently, in further studies, intraosseous markers can therefore be omitted.

In summary, the presented data demonstrate the effectiveness of ATFL suture tape augmentation in a cadaver experiment and the validity of the arthrometer to measure anterior talar drawer. This arthrometer therefore is recommended at least for quality management in the treatment of MAI patients and for preventive experimental and epidemiologic evaluations to further study CAI.

## Conclusions

Suture tape augmentation of the ATFL effectively protects the unstable anterolateral ankle in the sagittal plane. For additional CFL lesions, the stabilizing effect in the sagittal plane was reduced.

## Data Availability

The datasets used and/or analysed during the current study are available from the corresponding author on reasonable request.

## Abbreviations

ATFL: Anterior talofibular ligament
CAI: Chronic ankle instability
CFL: Calcaneofibular ligament
MAI: Mechanical ankle instability
PTFL: Posterior talofibular ligament
